# Association of coronary calcification with prognosis of Covid-19 patients without known heart disease

**DOI:** 10.1590/1414-431X2021e11681

**Published:** 2021-12-03

**Authors:** R.Y. Possari, H.J. Andrade-Gomes, V.C. Mello, E.A. Galdeano, L.F. Aguiar-Filho, M.S. Bittencourt, E.V. Ponte, L.R. Bertoche, L.R.S. Caio, J.D. Rodrigues, F.B. Alcantara, M.A.C. Freitas, J.C.G.C. Sarinho, N.K. Cervigne, W.M. Rodrigues, I. Aprahamian

**Affiliations:** 1 Departamento de Medicina Interna, Faculdade de Medicina de Jundiaí, Jundiaí, SP, Brasil; 2 Imagem Cardíaca, Prevent Senior, São Paulo, SP, Brasil; 3 Imagem Cardíaca, United Health Group, São Paulo, SP, Brasil; 4 Departamento de Medicina Interna, Hospital de Caridade São Vicente de Paulo, Jundiaí, SP, Brasil; 5 Instituto Dante Pazzanese de Cardiologia, São Paulo, SP, Brasil; 6 Centro de Pesquisa Clínica e Epidemiológica, Hospital Universitário, Universidade de São Paulo, São Paulo, SP, Brasil; 7 Laboratório DASA, São Paulo, SP, Brasil; 8 University of Pittsburgh Medical Center and University of Pittsburgh, Pittsburgh, PA, USA; 9 Departamento de Regulação da Saúde, Prefeitura de Jundiaí, Jundiaí, SP, Brasil; 10 Department of Psychiatry, University Medical Center Groningen, University of Groningen, Groningen, The Netherlands

**Keywords:** Covid-19, Coronavirus, Coronary artery calcification, Mortality, Respiratory failure

## Abstract

Risk factors that determine the severity of Covid-19 have not been fully elucidated. The aim of this study was to evaluate the role of coronary artery calcification (CAC) as a risk factor for death or mechanical ventilation (MV) of patients without known heart disease infected with Covid-19. We analyzed 283 consecutive in-patients with acute respiratory symptoms with chest computed tomography (chest-CT), without previous heart disease, and criteria for Covid-19 (RT-PCR positive and/or typical clinical and chest-CT findings). CAC was classified by the number of coronary segments affected as absent (0), mild (1-3), and severe calcification (more than 3). The association between CAC, CAC severity, and death or MV due to severe respiratory failure was assessed by logistic regression. The mean age was 58.7±15.7 years and 54.1% were men. Patients with CAC were older, more likely to have hypertension, and less likely to be obese. CAC was present in 75 patients (26.5%), of which 42 had a mild calcification and 33 had severe calcification, and was associated with death (OR=2.35, 95%CI: 1.01-5.48) or MV (OR=2.72, 95%CI: 1.20-6.20) adjusted for multiple confounders, with significant and increased odds ratio for the severe form of CAC (death: OR=3.70, 95%CI: 1.20-11.42; MV: OR=3.30, 95%CI: 1.09-9.95). We concluded that CAC was an independent risk factor for death or MV in Covid-19 patients without previous heart disease, particularly for those with severe calcification. CAC can be easily visualized on common chest-CT, widely used in evaluation of moderate to severe Covid-19.

## Introduction

Coronavirus disease 2019 (Covid-19) continues to have a great impact worldwide, being an important cause of morbidity and mortality, and has led to an increase in the incidence of cases and deaths in recent months in several countries (1). Despite the remarkable efforts of the scientific community to identify the factors associated with more severe cases, Covid-19 continues to surprise with critical illnesses and deaths in patients without relevant comorbidities or previous pathologies (2). Most cases are mild oligosymptomatic respiratory infections (3), although in moderate and severe cases, the typical clinical presentation is acute respiratory syndrome (SARS-CoV-2) in which cardiovascular disease is frequent (20-25%) and reported as an important determinant of poor clinical outcome (4,5). In Covid-19, cardiovascular disease can present as a preexisting comorbidity such as previous coronary heart disease and heart failure (6,7), or new events such as myocarditis (8,9), myocardial infarction (10), takotsubo cardiomyopathy (11,12), and right ventricular failure (13,14) caused by pulmonary embolism and pulmonary hypertension.

Coronary artery calcification (CAC) is a strong independent predictor of fatal and non-fatal events and is increasingly used for risk stratification in patients without heart disease (15,16). CAC can be detected on non-electrocardiogram gated low-dose chest computed tomography imaging (chest CT scan) (17-19), widely performed in moderate to severe SARS-CoV-2 patients. Recently, some studies reported heterogeneous results regarding the association between the presence of CAC and a composite outcome of death and invasive and non-invasive respiratory support for Covid-19 hospitalized patients (20-23), but these findings were contradictory in studies that excluded patients with previous heart disease.

We hypothesize that CAC, a subclinical form of cardiovascular disease (in patients without previously known coronary heart disease), is a determinant of poor prognosis in Covid-19 patients. Nonetheless, the aim of this study was to assess CAC as a risk factor for death or mechanical ventilation due to respiratory failure in Covid-19 patients without known heart disease.

## Material and Methods

### Design, participants, and procedures

**Figure 1:**
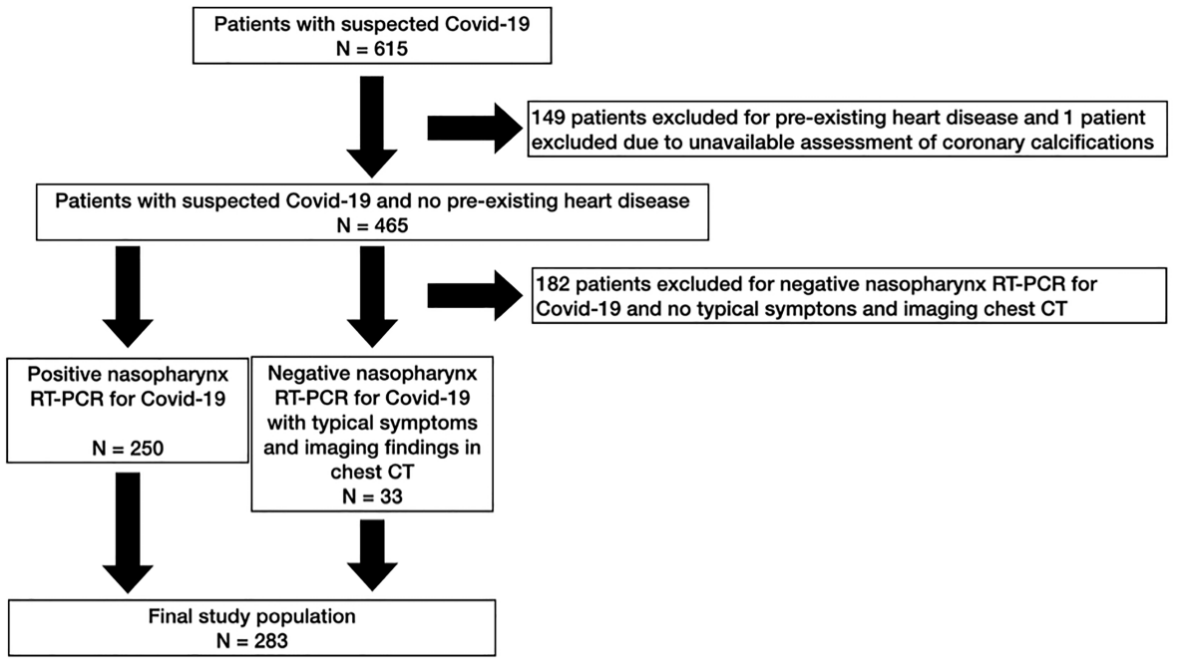
Flow diagram of participants in the study.

In this retrospective observational cohort study, data were collected from patients with clinically suspected Covid-19 infection between February and September 2020 at a university hospital in southwestern Brazil. Of a total of 615 patients admitted to the emergency department, 250 patients tested reverse transcription polymerase chain reaction (RT-PCR) for SARS-CoV-2 and 33 patients had typical Covid-19 symptoms and high-resolution imaging findings in chest computed tomography (initial RT-PCR negative) (Figure 1). Even with a negative RT-PCR result (inherent possibility of false negative or outside the RT-PCR positivity window), in the context of the pandemic, some patients were included in the study if they presented clinical signs compatible with acute respiratory infection and CT findings typical of Covid-19: ground-glass opacities and peripheral bilateral foci of consolidation, affecting multiple lung lobes, in the absence of masses, cavitated or not, and absence of pleural effusion (24,25). Individuals whose imaging exam was not performed with the proper quality technique (positioning and intensity of ionizing radiation) were excluded, as were patients who had previous heart disease (cardiomyopathy, heart failure, or coronary disease), described in the electronic medical record. The study complied with the Declaration of Helsinki and was approved by the Research Ethics Committee of the Faculty of Medicine of Jundiaí and also by the Research Ethics Committee of the Hospital de Caridade São Vicente de Paulo.

### Measurements

**Figure 2:**
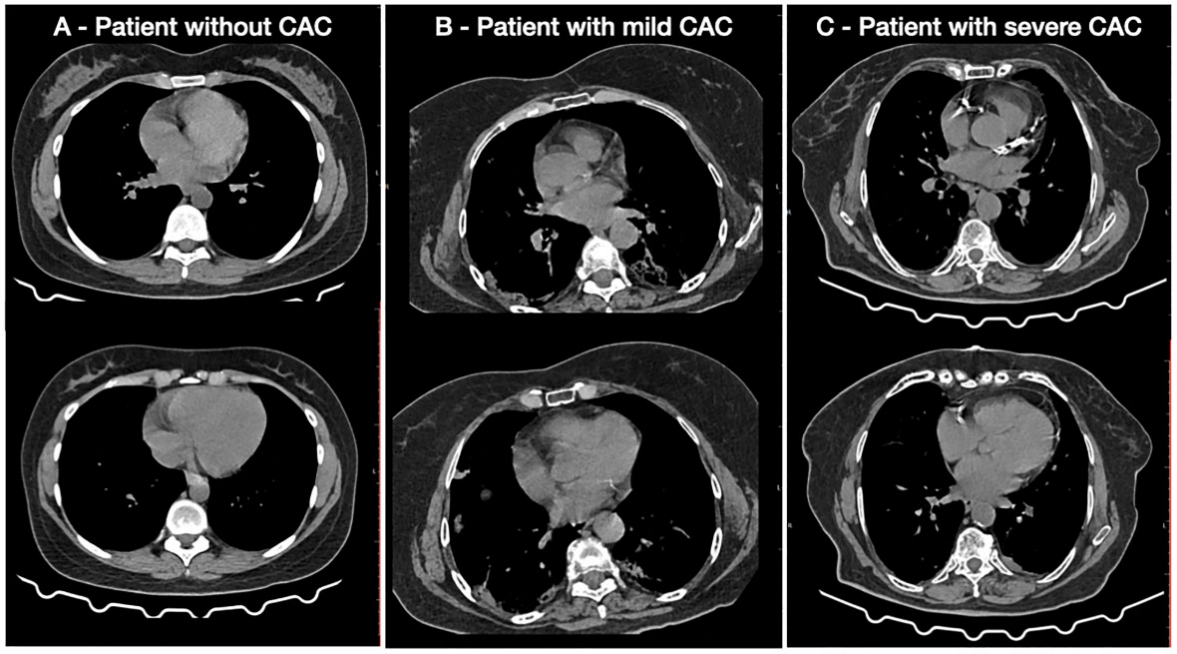
Coronary calcification in chest computed tomography scans. **A**, Examples of a patient without coronary artery calcification (CAC), **B**, a patient with mild CAC (presence of calcification in two coronary segments), and **C**, a patient with severe CAC (presence of calcification in six coronary segments).

Data on sociodemographic characteristics (age, gender), comorbidities (pre-existing diseases and concomitant heart disease), and clinical outcomes (length of hospitalization, need for mechanical ventilation, and death) were collected from electronic medical records. Chest CT scans were performed on Siemens Somatom Spirit Dual and Siemens Go UP 32 channels (Siemens Healthcare, Germany), respectively 1.0 and 0.8 s of gantry rotation time. Chest images were analyzed in slices of 1-mm thickness in high resolution acquisitions, in which the presence of CAC severity was evaluated and classified (17). For CAC scoring purposes, an ordinal scale of 0-9 was used based on the presence of calcification by the number of affected coronary segments as follows: left main coronary artery; anterior descending artery, proximal, middle, and distal thirds; circumflex artery, proximal and distal segments (or proximal, middle, and distal, if dominance is left); and right coronary artery, proximal, middle, and distal thirds (or proximal and distal, if dominance is left). Patients were classified as without calcification (0), having mild calcification (1-3), or having severe calcification (>3) (Figure 2) (17). The CAC analysis was performed by a cardiologist with 8 years of experience as a cardiac imaging specialist (H.J.A.-G.) and blinded to the clinical characteristics and evolution of included patients, except if the patient was intubated at the time of CT scan (because the orotracheal tube is visible).

### Statistical analysis

Baseline characteristics of samples are reported as categorical (percentages) and continuous (mean and standard deviation or median and 25-75 quartiles) measures. All continuous variables had a non-parametric distribution after histogram analysis and Shapiro-Wilk test, except for age and temperature. Chi-squared test (or Fisher's test if 5 or less patients were included in the variable) and Mann-Whitney test were used to compare categorical and continuous variables, respectively, between Covid-19 patients with and without CAC. Multiple adjusted binary logistic regression models were used to evaluate the association between CAC, CAC severity (none, mild, and severe), and death or mechanical ventilation due to severe respiratory failure. Additionally, a sensitivity analysis was performed only for the RT-PCR-positive cases of Covid-19. Goodness-of-fit was tested through Akaike Information Criterion (AIC) values, Hosmer-Lemeshow test, and *R^2^* (Cox-Snell). Age, male gender, dyspnea, hypertension, diabetes, obesity, and lymphocyte count were confounders in the logistic regression model, as well as C-reactive protein (CRP) and D-dimer, available in 68 and 76% of patients, respectively. These variables were chosen based on previously published studies identifying them as risk factors for worse prognosis (26). Age was used as a quadratic term because of its non-linear relationship with Covid-19 (27,28). P-values less than 0.05 were considered statistically significant. Data were analyzed using Statistical Package of the Social Sciences (SPSS, IBM, USA), version 25.0.

## Results

Of the 615 patients admitted to the emergency department due to acute respiratory syndrome, 283 fulfilled the criteria for Covid-19 infection. Mean age was 58.7±15.7 years old and 54.1% were men. The mean time from first symptom of infection to hospital admission was 7.2±4.5 days. At admission, the mean temperature, systolic blood pressure, diastolic blood pressure, and oximetry were 38.1±0.77°C, 130.8±24.2 mmHg, 79.2±14.5 mmHg, 89.2±6.3%, respectively. Median and interquartile values of lymphocyte count, CRP, and D-dimer were 1,120 (720-1,750) cells, 11.3 (4.9-22.6) mg/dL, and 1.414 (825.5-3343.3) ng/mL, respectively.

Baseline characteristics of the sample are presented in Table 1, comparing patients with and without CAC (Table 1). Covid-19 patients with CAC (n=75) were older, were more likely to have hypertension, and less likely to be obese. CAC was found in 75 patients (26.5%), of whom 42 showed a score from 1-3 (mild) and 33 had severe presentation (score >3). Of the sample with Covid-19, mechanical ventilation due to severe respiratory failure occurred in 99 (35%) patients and death in 94 (33.2%). Forty (53.3%) patients with CAC died and 35 (46.7%) received mechanical ventilation for respiratory support, while among patients without CAC, 54 (30%) died and 64 (30.8%) required mechanical ventilation, (respectively, P<0.001 and P=0.013).

**Table 1:** Baseline characteristics of the sample with Covid-19.

Characteristics	Coronary calcification (n=75)	No coronary calcification (n=208)	P-value
Age (years), mean (SD)	71.4 (9.4)	54.2 (15.0)	<0.001^1^
Male gender, n (%)	44 (58.7)	125 (60.1)	0.829
Fever, n (%)	37 (49.3)	128 (61.5)	0.066
Cough, n (%)	57 (76.0)	158 (76.0)	0.995
Dyspnea, n (%)	52 (69.3)	156 (75.0)	0.340
Anosmia, n (%)	6 (8.0)	15 (7.2)	0.823
Sore throat, n (%)	1 (1.3%)	8 (3.8)	0.453
Runny nose, n (%)	10 (13.3)	19 (9.1)	0.304
Gastrointestinal symptoms, n (%)	7 (9.3)	16 (7.7)	0.656
Fatigue, n (%)	8 (10.7)	19 (9.1)	0.699
Myalgia, n (%)	16 (21.3)	66 (31.7)	0.089
Hypertension, n (%)	54 (72.0)	88 (42.3)	<0.001
Diabetes, n (%)	28 (37.3)	53 (25.5)	0.052
Obesity, n (%)	10 (13.3)	51 (24.5)	0.043
COPD, n (%)	6 (8.0)	8 (3.8)	0.155
Renal disease, n (%)	4 (5.3)	7 (3.4)	0.489
Lymphocyte, median (IQR)	1065 (580, 1565)	1025 (745, 1525)	0.403^2^
CRP, median (IQR)	14.7 (7.4, 24.0)	14.2 (5.3, 23.6)	0.163^2^
D-dimer, median (IQR)	2567.8 (1144.7, 8058.1)	1192.1 (753.3, 2151.9)	<0.001^2^

Chi-squared test for categorical variables; ^1^ *t*-test; ^2^Mann-Whitney test. COPD: chronic obstructive pulmonary disease; IQR: interquartile range; CRP: C-reactive protein.

CAC was associated with death or mechanical ventilation after adjustment for age (OR=4.38, 95%CI: 2.63-7.30, P<0.001 for death; OR=2.54, 95%CI: 1.54-4.19, P<0.001 for mechanical ventilation) and multiple confounding factors (OR=2.35, 95%CI: 1.01-5.48, P<0.047 for death; OR=2.72, 95%CI: 1.20-6.20, P<0.017 for mechanical ventilation). When adjusted for age, both the presence and severity of CAC were associated with death or mechanical ventilation. In addition, the odds ratio increased from the mild (OR=3.95 95%CI: 2.08-7.50 for death; OR=2.20, 95%CI: 1.18-4.10 for mechanical ventilation) to the severe form of CAC (OR=4.73, 95%CI: 2.37-9.45 for death; OR=2.63, 95%CI: 1.35-5.10 for mechanical ventilation). A sensitivity analysis of RT-PCR-positive Covid-19 cases did not result in significantly different findings (Table 2). When adjusted by multiple confounding factors, severe CAC maintained the association with the need for mechanical ventilation (OR=3.30, 95%CI: 1.09-9.95) and death (OR=3.70, 95%CI: 1.20-11.42). These findings are summarized in Table 3 (age-adjusted) and Table 4 (adjusted for multiple confounding factors).

**Table 2:** Sensitivity analysis of the association between coronary calcification and death or mechanical ventilation due to respiratory failure adjusted by age among RT-PCR-positive Covid-19 cases.

	Death			Intubation		
	OR	95%CI	P	OR	95%CI	P
Coronary calcification	4.46	2.58, 7.70	<0.001	2.49	1.47, 4.22	<0.001
Mild coronary calcification^1^	4.36	2.17, 8.78	<0.001	2.29	1.18, 4.45	0.015
Severe coronary calcification^1^	4.29	2.06, 8.92	<0.001	2.34	1.17, 4.70	0.017

Binary logistic regression; ^1^absence of calcification was the reference for analysis.

**Table 3:** Association between coronary calcification and death or mechanical ventilation due to respiratory failure adjusted by age.

	Death			Intubation		
	OR	95%CI	P	OR	95%CI	P
Coronary calcification	4.38	2.63, 7.30	<0.001	2.54	1.54, 4.19	<0.001
Mild coronary calcification^1^	3.95	2.08, 7.50	<0.001	2.20	1.18, 4.10	0.013
Severe coronary calcification^1^	4.73	2.37, 9.45	<0.001	2.63	1.35, 5.10	0.040

Binary logistic regression; ^1^absence of calcification was the reference for analysis.

**Table 4:** Association between coronary calcification and death or mechanical ventilation due to respiratory failure adjusted by multiple confounders.

	Death			Intubation		
	OR	95%CI	P*	OR	95%CI	P*
Coronary calcification	2.35	1.01, 5.48	0.047^a^	2.72	1.20, 6.20	0.017^b^
Mild coronary calcification^1^	1.94	0.68, 5.50	0.213	2.70	0.98, 7.47	0.055
Severe coronary calcification^1^	3.70	1.20, 11.42	0.023^c^	3.30	1.09, 9.95	0.033^d^

Binary logistic regression; *adjusted by age (quadratic term), male gender, dyspnea, hypertension, diabetes, obesity, log (lymphocyte count), log (CRP), and D-dimer; ^1^absence of calcification was the reference for analysis. ^a^Dyspnea (P=0.041) and obesity (P=0.031), ^b^log(CRP), ^c^obesity (P=0.025) and D-dimer (P=0.039), and ^d^log(CRP) were also significant independent variables.

## Discussion

In the present study, CAC was associated with death and mechanical ventilation due to severe respiratory failure in Covid-19 patients without previous heart disease. Specifically, mortality rate in our cohort was 33.2% in general and 53.3% among CAC-positive patients. Moreover, the severity of CAC was significant regarding this association.

Cardiac manifestations and outcomes related to Covid-19 are still under investigation. CAC is an important subclinical marker for future cardiovascular events in asymptomatic individuals (15,19) and was the main target of this study, demonstrating a risk factor independent of other biomarkers (lymphocytopenia, systemic artery pressure, CRP, and elevated D-dimer) (28,29) already associated to a risk for orotracheal intubation and mortality in hospitalized patients with Covid-19.

Our results confirmed those previously published by Dillinger and colleagues (20) who observed an association between the presence of CAC and a composite outcome consisting of mechanical noninvasive or invasive ventilation, extracorporeal membrane oxygenation, or death in patients without previous cardiovascular disease. However, in their study, mortality did not differ significantly between CAC-positive and -negative patients (20). Other studies (21-23) have consistently reported that survivors of SARS-CoV-2 had less coronary calcification than patients who died, although these studies did not exclude those patients with previous heart disease, a widely known factor of poor prognosis.

Furthermore, our study showed that the more extensive and severe the calcification the greater the chance of death or intubation compared with mild calcification. The presence of CAC increased the chance of death by 2.35 times, and an important calcification in more than three coronary segments, the odds ratio increased to 3.70.

Coronary calcification on chest CT of these patients, in addition to serving as an early diagnosis of coronary artery disease (condition already reported as a risk factor for Covid-19 severity and fatality), could represent some degree of biological fragility and a more accurate vascular aging index than the age of the patient.

Our study had several strengths that must be highlighted. We analyzed a similar number of patients to the previously mentioned studies in patients without heart disease (20,21), and death and mechanical ventilation due to severe respiratory failure were considered independent outcomes. Moreover, both the presence and severity of CAC were associated with worst prognosis. Finally, our findings were adjusted for multiple important confounders including age, comorbidities, and biomarkers predictive of worse prognosis, and goodness-of-fit was tested for accuracy of the model. Age was considered a quadratic term due to its non-linear relation to Covid-19 epidemiology (27,28).

This study should be read within the context of its design. The use of two different CT scanners can determine differences in accuracy for the assessment of coronary calcifications. In addition, the method used to quantify calcification was not the most recommended and used in clinical practice (ECG-gated), but it is an alternative method that can be useful, since many of the hospitalized patients suspected for SARS-CoV-2 end up undergoing high-resolution chest CT scan. As for the number of patients, a larger sample size, especially with more high-risk patients with important calcification, could bring more information and perhaps attribute significant statistical value to the extent of coronary calcification in relation to clinical outcomes. Moreover, in-depth details such as medications in use (e.g., statins) and time to death or mechanical ventilation could reinforce the evidence of our findings. The population included in this study was referred for hospitalization and chest tomography, as indicated by the physicians, to the only reference public hospital in the region, which is generally characterized by high mortality from the disease, not reflecting the reality of infected patients in general. For example, in Brazil, the mortality rate has been 2.7% (30) during acquisition of these data, which was similar to other countries (31). Additionally, we did not collect data regarding the severity of pulmonary involvement in chest CT. Finally, the observational nature of the present study does not allow inferring causality and does not exclude the existence of residual confounding risk factors.

In the present study, the existence of CAC observed on high-resolution chest-CT scan was an independent risk factor for death in patients with Covid-19 and unknown heart disease. In addition, our study showed that extension of calcification to more than 3 segments was associated with a worse prognosis than mild calcification or absence of coronary calcification. Therefore, chest-CT scan proved to be a simple and practical method for detecting CAC, and it is widely available in patients for diagnostic evaluation of Covid-19.
